# Uncovering the mystery of genetic heterogeneity in inherited peripheral neuropathies

**DOI:** 10.1093/lifemedi/lnad026

**Published:** 2023-07-07

**Authors:** Chanjuan Huo, Qinqin Cui, Ge Bai

**Affiliations:** Department of Neurobiology and Department of Neurology of Second Affiliated Hospital, Zhejiang University School of Medicine, Hangzhou 310058, China; Liangzhu Laboratory, MOE Frontier Science Center for Brain Science and Brain-machine Integration, State Key Laboratory of Brain-machine Intelligence, Zhejiang University, Hangzhou 311121, China; NHC and CAMS Key Laboratory of Medical Neurobiology, Zhejiang University, Hangzhou 310058, China; Department of Neurobiology and Department of Neurology of Second Affiliated Hospital, Zhejiang University School of Medicine, Hangzhou 310058, China; Liangzhu Laboratory, MOE Frontier Science Center for Brain Science and Brain-machine Integration, State Key Laboratory of Brain-machine Intelligence, Zhejiang University, Hangzhou 311121, China; NHC and CAMS Key Laboratory of Medical Neurobiology, Zhejiang University, Hangzhou 310058, China; Department of Neurobiology and Department of Neurology of Second Affiliated Hospital, Zhejiang University School of Medicine, Hangzhou 310058, China; Liangzhu Laboratory, MOE Frontier Science Center for Brain Science and Brain-machine Integration, State Key Laboratory of Brain-machine Intelligence, Zhejiang University, Hangzhou 311121, China; NHC and CAMS Key Laboratory of Medical Neurobiology, Zhejiang University, Hangzhou 310058, China

Marked genetic heterogeneity is correlated with the causation of many complex diseases, in which the similar phenotypes are often caused by different genetic mechanisms. For example, rare germline mutations in *P53*, *CHEK2*, and several other genes are associated with an increased risk of breast cancer. Each of these genes has been found to operate in networks integral to DNA repair and genomic integrity, suggesting that the genetic heterogeneity of this disorder is likely mediated by genes in related pathways. However, for many human diseases, it remains unclear whether there are such molecular links underlying their genetic heterogeneity.

Charcot-Marie-Tooth (CMT) diseases are the most common inherited peripheral neuropathies in the clinic. CMT2 is an axonal form of CMT, which leads to the peripheral axon degeneration and motor deficits in patients. CMT2 can be further classified into over 30 different subtypes based on their causing genes. Despite their similar clinical symptoms, these CMT2-causing proteins exhibit a great diversity in their cellular localizations and functions. The molecular mechanism underlying their genetic heterogeneity was poorly understood. Identification of their potential molecular links represents a critical step toward developing uniform treatment for different subtypes of the disease. In the recent issue of *Cell*, Cui *et al.* used CMT2D-causing mutant protein, glycyl-tRNA synthetase (GlyRS), as the entry point to identify a stress granule (SG)-related pathogenic pathway, which may represent a mechanistic link shared by many other CMT2 subtypes [1].

Neurological disorders often involve interplays between genetic predisposition and environmental stressors. SG formation is an important defensive mechanism in response to environmental insults. SGs can help to avoid protein mistranslation, and effectively organize various signaling molecules and energy resources in cells, thereby improving cell survival upon stress. In this study, Cui *et al*. found that, upon exposure to environmental stressors, GlyRS adopted a new cellular location in SGs, distinct from its normal cytoplasmic location. In SGs, mutant GlyRS perturbed the G3BP-centric core SG network by aberrantly binding to G3BP, causing the over-sequestration of non-SG molecules in SGs, thereby disturbing the cellular stress response and leading to an increased stress-vulnerability in motor neurons. Disrupting their aberrant interaction eliminated the impact of mutant GlyRS on SGs, improved the stress vulnerability of motor neurons, and alleviated motor deficits in CMT2D mouse models. Moreover, a similar mechanism was largely shared by many other CMT2 subtypes. In this study, a combination of approaches was employed, including mouse genetics, neuropathology, live-cell imaging, proximity labeling, quantitative MS-based proteomics, super-resolution imaging (STORM), and so on [1].

This work identified SG abnormality as an important pathogenic mechanism underlying CMT pathogenesis and provided a new clue for understanding the genetic heterogeneity of complex diseases. There are also several intriguing points inspired by this study:

## The interplay between genetic and environmental pathways in CMT pathogenesis

Over the past decades, an increasing number of chemical and physical insults, such as chemotherapy drugs, heavy metals, toxins, and radiation, have been identified to predispose peripheral nerves to neuropathy. These stressors contribute to the activation of various stress pathways including oxidative stress, mitochondrial stress, endoplasmic reticulum (ER) stress, and others. In addition to these environmental insults, pathophysiological stresses may also play important roles in CMT pathogenesis. In this study, Cui *et al*. identified that high-intensity exercise may act as one of these important physiological stressors, contributing to the progression of peripheral neuropathology in pre-symptomatic CMT2D mice [1]. Clinical observations show that the dominant hands of CMT patients are often weaker than the nondominant hands, as correlated with their frequency of use, which may be attributable to similar factors [2].

Despite the CMT-related stressors differing considerably in their associated stress pathways, there seems to be a trend in that they are consistently accompanied by SG formation in cells. In comparison, AZE, TCM, and other stressors that do not induce SG formation seem far less potent to CMT2D motor neurons. These findings linked CMT pathogenesis to SG-related mechanisms. They further found that GlyRS^CMT2D^ could aberrantly bind to G3BP and strengthen the G3BP-centric core SG network, leading to the aberrant sequestering of non-SG signaling molecules into SGs. Since SGs represent an important anti-stress signaling hub in the cell, SG network abnormalities result in dysregulation of cellular stress responses, thereby contributing to increased stress vulnerability in motor neurons. In this way, the CMT2D-causal genetic pathway and environmental stress pathways are integrated into the aberrant molecular interplay between GlyRS^CMT2D^ and G3BP proteins in SGs [1].

It is worth noting that SG abnormalities do not appear to be the initiating cause of CMT neuropathy, as no obvious SGs were observed in CMT2D mouse models before the disease onset. Interestingly, recent studies from Burgess Lab have found that the integrated stress response (ISR) was activated in CMT2D mice by 1–2 weeks of age [3]. Considering SG formation is an important downstream event of ISR, it may suggest that CMT2D neuropathy is initiated by activation of ISR, then further aggravated by deficient stress responses associated with SG abnormalities upon exposure to environmental/pathophysiological stressors.

## Hierarchic organization of multiple pathogenic mechanisms underlying CMT2 neuropathies

CMT2D neuropathy is characterized by distal muscle weakness due to motor axon loss. Considering GlyRS proteins are broadly expressed and required in all cells, it is an intriguing question how GlyRS^CMT2D^ selectively harms distal motor axons. Previous findings reported that the neomorphic binding of GlyRS^CMT2D^ to Nrp1 blocked VEGF/Nrp1 signaling, an essential neurotrophic factor for motor neuron survival, thus contributing to the motor neuron vulnerability in CMT2D [4]. Another study found that the aberrant binding of GlyRS^CMT2D^ to HDAC6 promoted its deacetylase activity and impaired α-tubulin acetylation, which causes axonal transport deficits in peripheral nerves. This mechanism provides an explanation for the vulnerability of distal axons in CMT2D [5]. As a result, the combination of these cellular deficiencies makes distal motor axons the most vulnerable part of the body under stress conditions. Meanwhile, ISR has been found to be mainly activated in motor neurons [3]. All of these observations suggest a hierarchical model of multiple pathogenic mechanisms contributing to the clinical presentations of CMT2D neuropathies. A similar principle may also be applied to understanding the multiple pathogenic mechanisms underlying other CMT subtypes.

## Mechanistic links among different CMT2 subtypes

**Figure 1.**
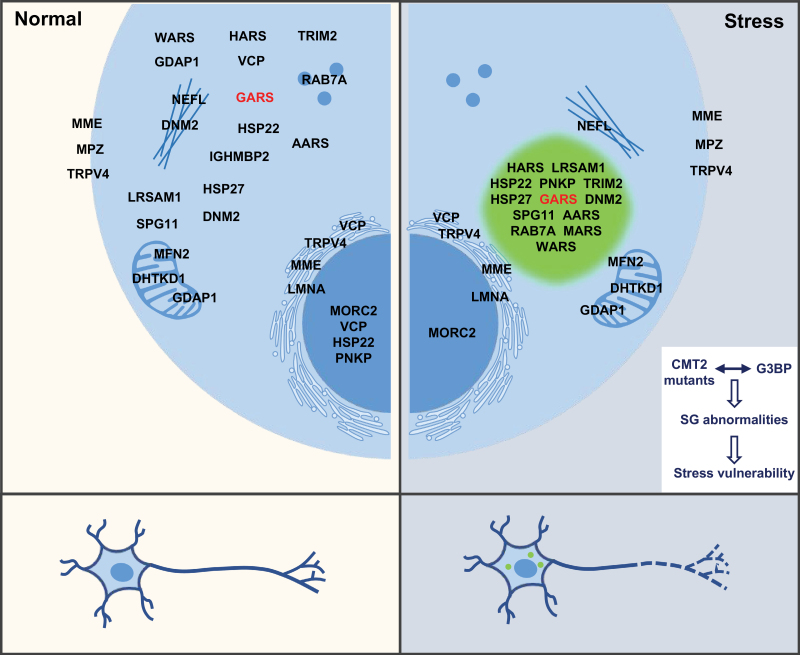
**Schematic depicting a stress-dependent molecular link underlying the genetic heterogeneity of CMT2 neuropathies.** For CMT2, similar peripheral neuropathology is caused by over 30 different mutant proteins with various subcellular localizations and functions (left panel). Environmental stressors induce these CMT2-associated proteins to redistribute to an SG-associated localization pattern (right panel). Moreover, GlyRS^CMT2D^ and other CMT2 mutant proteins can aberrantly interact with G3BP1 and perturb the G3BP-centric core SG network, ultimately leading to an increased stress vulnerability in motor neurons. This model provides a conceptual framework for understanding the genetic heterogeneity of CMT2 neuropathies with environmental stress-oriented aspects.

Genetic heterogeneity is a hallmark of many complex diseases. In Charcot-Marie-Tooth type 2 diseases (CMT2), similar peripheral neuropathology is inexplicably caused by over 30 different mutated genes. In this study, Cui *et al*. found various CMT2 proteins adopted similar properties upon stress, which link them to SG-related mechanisms. More specifically, Cui *et al*. found that, upon stress, most of the tested CMT2 mutant proteins entered SGs and aberrantly interacted with G3BP. This strengthened the SG network and led to increased stress vulnerability in motoneurons. It is worth noting that a few CMT2 proteins were not located in SGs upon stress but remained in mitochondria, ER, or other organelles. Since these organelles have been associated with the regulation of SG localization and function, it raised a possibility that these CMT proteins may influence SGs through inter-organelle interactions (Fig. 1) [1].

In summary, this work provides a conceptual framework for understanding the genetic heterogeneity of complex diseases with stress-oriented aspects and uncovers a SG-associated molecular interplay model between genetic and environmental pathways. It also expands our understanding on how disease proteins interfere with the function of membraneless organelles that can then lead to neurodegeneration independent of forming protein aggregates. In light of the work presented here, future studies should focus on identifying SG-targeted drugs, which could pave the way toward developing uniform treatments for these rare diseases.
